# Biodegradation of waste lubricants by a newly isolated *Ochrobactrum* sp. C1

**DOI:** 10.1007/s13205-015-0282-9

**Published:** 2015-03-03

**Authors:** Munna Bhattacharya, Dipa Biswas, Santanu Sana, Sriparna Datta

**Affiliations:** Department of Chemical Technology, University of Calcutta, 92, A.P.C Road, Kolkata, 700009 West Bengal India

**Keywords:** Biodegradation, Hydrocarbons, Waste engine oil, Waste transformer oil, Emulsification index

## Abstract

A potential degrader of paraffinic and aromatic hydrocarbons was isolated from oil-contaminated soil from steel plant effluent area in Burnpur, India. The strain was investigated for degradation of waste lubricants (waste engine oil and waste transformer oil) that often contain EPA (Environmental Protection Agency, USA) classified priority pollutants and was identified as *Ochrobactrum* sp. C1 by 16S rRNA gene sequencing. The strain C1 was found to tolerate unusually high waste lubricant concentration along with emulsification capability of the culture broth, and its degradation efficiency was 48.5 ± 0.5 % for waste engine oil and 30.47 ± 0.25 % for waste transformer oil during 7 days incubation period. In order to get optimal degradation efficiency, a three level Box–Behnken design was employed to optimize the physical parameters namely pH, temperature and waste oil concentration. The results indicate that at temperature 36.4 °C, pH 7.3 and with 4.6 % (v/v) oil concentration, the percentage degradation of waste engine oil will be 57 % within 7 days. At this optimized condition, the experimental values (56.7 ± 0.25 %) are in a good agreement with the predicted values with a calculated *R* ^2^ to be 0.998 and significant correlation between biodegradation and emulsification activity (*E*_24_ = 69.42 ± 0.32 %) of the culture broth toward engine oil was found with a correlation coefficient of 0.972. This is the first study showing that an *Ochrobactrum* sp. strain is capable of degrading waste lubricants, which might contribute to the bioremediation of waste lubricating oil-contaminated soil.

## Introduction

The waste lubricating oil, otherwise called spent oil or used-lubricant, obtained after servicing and subsequent draining from automobiles, generators and industrial machines, is disposed off indiscriminately all over the world, and adequate attention has not been given to its disposal (Bartz 2006). This waste oil often contains appreciable amounts of EPA priority pollutants such as toxic heavy metals, polyaromatic hydrocarbons (PAHs), chlorinated hydrocarbons (PCBs) and other hydrocarbon contaminants that are known to have carcinogenic and neurotoxic effects on biological system (Ray et al. 2008; Chandra et al. 2013). Used oils can be re-refined to base lube oil by proper recycling process. But high contamination levels in waste oils increase the difficulties in recycling operation and as a result uncontrolled dumping in soil and drains and land filling of waste oils lead to environmental hazards with global implications (Misra and Pandey 2005; Lin et al. 2010). For environmental protection and to minimize pollution level, most of the industries follow the conventional thermal destruction process or acid-clay process to degrade the waste oils (USEPA 2004). But these are expensive, time-consuming and not environmentally safe procedures (Das and Chandran 2011). Bioremediation is the method of choice now-a-days due to its cost-effectiveness and wide environmental acceptability (Singh et al. 2009) that is based on simple microbial degradation phenomena by which complete removal of hydrocarbon pollutants can be done by using microorganisms (Gómez et al. 2007). Moreover, the ability of producing surface-active agents such as biosurfactants/bioemulsifiers is of major importance (Batista et al. 2006; Banat et al. 2010) that are directly involved in the process of hydrocarbon biodegradation from the environment through increased bioavailability and subsequent uptake of hydrophobic compounds by direct cell contact (Franzetti et al. 2010).

The base lubricating oil contains long chain (C_16_–C_36_) saturated hydrocarbons and more than 75 % cyclic alkanes (Koma et al. 2003) that are known as recalcitrant to microbial degradation. In addition, the presence of toxic metals, PAHs, etc. in used oil inhibits the microbial degradation process (Adesodun and Mbagwu 2008; Lee et al. 2008; Rauchyte et al. 2006). So, it is challenging to isolate a single microorganism having degrading potentials of diverse constituents of the base lube oil and effective for significant degradation of waste oils (Adebusoye et al. 2007; Ghazali et al. 2004). The microorganisms mostly isolated from hydrocarbon contaminated sites, having waste lubricating oil degrading ability, belong to the genera *Pseudomonas*, *Rhodococcus*, *Alcaligenes*, *Acinetobacter*, *Arthrobacter*, *Citrobacter*, *Serratia*, *Micrococcus*, *Bacillus*, etc. (Batista et al. 2006; Ron and Rosenberg 2014). Several results have been reported on biodegradation of petroleum hydrocarbons of waste motor lubricating oil-contaminated soils in recent times (Abioye et al. 2012; Aleer et al. 2011; Jain et al. 2010; Adesodun and Mbagwu 2008). But there are no such reports till date on waste lubricating oil degradation by *Ochrobactrum* sp. to the best of our knowledge.

Hence, our aim is to degrade the diverse range of hydrocarbons present in waste lubricating oil samples e.g., waste engine oil (WEO) and waste transformer oil (WTO) by an isolated culture of *Ochrobactrum* sp. C1, followed by the study of degradation efficiency of the strain and potential for surface-active compound production. Since biodegradation of waste oil is a natural process limited by several physicochemical factors (Chandra et al. 2013), optimization of the culture conditions has been carried out by response surface methodology (RSM) for enhanced biodegradation and a correlation between degradation and emulsification capability of the culture broth has also been established by this study.

## Materials and methods

### Chemicals

WEO (specific gravity-0.86) and WTO (specific gravity-0.815) were collected from local automobile workshops and power generating stations nearby Kolkata, West Bengal, India. Fresh engine oil, transformer oil, gear oil, brake oil, diesel, kerosene were procured from retail market, and pure hydrocarbons were procured from Sigma Aldrich Co. USA. Other chemicals and solvents were of AR grade and purchased from Merck Co. Germany. Bushnell-Haas (BH) media and Nutrient agar media of Hi-Media Laboratories Pvt. Ltd were used for isolation, cultivation and maintenance of culture.

### Isolation and selection of microorganism

Bushnell-Haas (BH) media with the following composition (g/L): K_2_HPO_4_ (1.0 g), KH_2_PO_4_ (1.0 g), NH_4_NO_3_ (1.0 g), MgSO_4_·7H_2_O (0.2 g), FeCl_3_·6H_2_O (0.05 g), CaCl_2_·2H_2_O (0.02 g), was used as enrichment medium with WEO as the sole carbon source to isolate waste lubricating oil degrading bacteria. Oil-contaminated soil samples were collected from 24 different locations including local automobile workshops, petroleum industry effluent area, and steel plant effluent area in and around Kolkata, India. Soil samples (~10 gm) were added to 50 ml BH media taken in 250-ml Erlenmeyer culture flasks with 2 % (v/v) waste oil and incubated at 37 °C at 100 rpm in a rotary shaker incubator (ORBITEK-LJE, Scigenics Biotech Pvt. ltd., Chennai, India) for 7 days. After 7 days incubation, the cultures were isolated as single colony on to Nutrient Agar (NA) media by streak-plate method. They were maintained in slant cultures by preserving at 4 °C and subculturing at 2 weeks interval.

For selection of microorganism, the isolated cultures were screened for effective waste lubricating oil degrader as well as surface-active agent producer. Fresh overnight cultures (OD_600_ = 1.0) suspended in BH media were used as inoculum 2 % (v/v) for all the experiments and inoculated aseptically in culture flasks with 2 % (v/v) carbon source at same culture conditions. After completion of the incubation period, the culture broth samples were centrifuged at 5,000 rpm (REMI R24) for 20 min and the culture supernatant was separated from the oil phase in a separating funnel. The culture supernatant was then evaluated for surface activity by measuring surface tension and emulsification index. The residual oil was measured for evaluating the degradation efficiency of the isolated microorganism. All the experiments were performed in triplicate, and a control devoid of the bacterial isolates was prepared for each set of experiments.

### Identification of the selected microorganism C1

The genomic identification of the selected bacterial isolate was carried out by 16S rRNA gene sequencing method from Bhat Biotech India Pvt. Ltd. Bangalore, India. DNA extraction was done using genomic DNA extraction Kit (Bhat Biotech), and PCR amplification of the 16 s rRNA gene was performed using the universal primers. The PCR products were sequenced by automated DNA sequencer—3037*xl* DNA analyzer from Applied Biosystems using BigDye^®^ Terminator v3.1 cycle sequencing Kit (Applied Biosystems). Sequence data were aligned, and dendrograms were generated using Sequence analysis software version 5.2 from Applied Biosystems. Sequences were compared to the non-redundant NCBI database by using BLASTN, to find the most similar sequence, sorted by the E score. A representative sequence of 10 most similar neighbors was aligned using CLUSTAL W2 for multiple alignments with the default settings. The multiple-alignment file was then used to create phylogram using MEGA5 software.

### Biodegradation studies

#### Determination of degradation ability on different hydrocarbons

Several hydrocarbons of naphthene–paraffin–aromatic (N–P–A) series like nonane, dodecane, tetradecane, hexadecane, octadecane, eicosane, octacosane, decalin, tetralin, xylene, naphthalene, phenanthrene and anthracene were tested with the bacterial isolate to study its degradation ability of the type and range of hydrocarbons. Various petroleum fractions like lubricating oil base stock (LOBS), vacuum gas oil (VGO), diesel, kerosene and fresh lubricating oils like engine oil, transformer oil, brake oil, gear oil were also tested with the bacterial isolate to establish its degradation ability of different petroleum fractions from low boiling kerosene to high-boiling lubricating oils. All the hydrocarbons tested were at 2 % (v/v) concentration level at same culture parameters mentioned above.

#### Determination of degradation ability on waste lubricating oils with varying culture conditions

Waste lubricating oils namely WEO and WTO were treated with culture condition variations such as, incubation period (7, 14 and 21 days), pH (5, 7 and 9), incubation temperature (32, 37, and 42 °C) and oil concentration (2, 5 and 10 % v/v) to study the effect on waste oil percent degradation ability of the culture.

### Biodegradation analysis

#### Bacterial growth determination

The biomass concentration in the culture broth was determined by dry weight method. In this method, the broth was centrifuged at 5,000 rpm for 20 min. The bacterial mass was then transferred to a pre-weighted aluminum cup and dried at 50 °C overnight. The exact weight of the bacterial mass was determined by subtracting the weight of dry cup from that of the cup containing dry bacterial mass. The cell concentrations were also determined using spectrophotometer at 600 nm.

#### Hydrocarbon analysis using gas chromatography

The residual oil sample from inoculated and un-inoculated culture flasks was extracted three times with hexane after each biodegradation experiment according to Adebusoye et al. (2007). The organic phase was concentrated by evaporation of the solvent after drying over anhydrous Na_2_SO_4_ and analyzed by gas chromatography according to the condition described by Ghazali et al. (2004). Hexane extracts of residual oil sample (1 µl) were injected for analysis by using a Polaris Q Mass Spectrometer coupled with Thermo Scientific Trace 1300 series gas chromatograph and TR-5 column (30 × 10^3^ cm length; 0.032 cm id; and 1 × 10^−3^ cm film thickness). Nitrogen was used as carrier gas. The injector and detector temperatures were maintained at 300 and 280 °C, respectively. The oven was programmed at an initial temperature of 40 °C; this was held for 2 min, then ramped at 15 °C/min to 300 °C and held for 10 min. The relative percent degradation of WEO and WTO was calculated by the differences in summation of peak area of total petroleum hydrocarbons (TPH) present in the residual oil compared to that from un-inoculated control flasks (Bhattacharya and Biswas 2014a). Degradation efficiency of pure hydrocarbons was also calculated by the differences in summation of peak area compared to their respective standards. Chromatographs were analyzed by Chromeleon 7.0 program and a library (NIST 2007) search was performed for identification of chromatogram peaks.

#### Surface tension measurement

Surface tension of cell-free and oil-free culture supernatant was measured by the application of a digital tensiometer (Dataphysics DCAT 11, Germany) at 30 °C using du Nouy ring method (Lunkenheimer and Wantke 1981).

#### Emulsification index measurement

Emulsification index (*E*_24_) was measured following the method described by Cooper and Goldenberg (1987). Two milliliters of engine oil was added to 2 ml of cell-free extract and vortexed at high speed for 2 min. Diesel, kerosene, crude oil and transformer oil were also used as substrate for emulsification. Measurements were taken after 24 h as follows:

$$ E_{ 2 4} = \, \left( {{\text{height of the emulsion}}/{\text{total height}}} \right) \, \times { 1}00 $$

### Optimization studies using response surface methodology

Preliminary trials indicated that pH, temperature and waste oil concentration in the culture medium were the significant variables for the percentage biodegradation of waste oils. Hence, pH, temperature and waste oil concentration were chosen as the three variables for optimization studies. The three levels of each variable are given in Table 1. Percent degradation of WEO and *E*_24_ values was taken as two responses.

**Table 1:** Box–Behnken design for the three independent variables on biodegradation of waste engine oil and corresponding *E*_24_ of the culture broth

Runs	*X*_1_ (temperature °C)	*X*_2_ (pH)	*X*_3_ (oil conc. v/v %)	*E*_24_ value (%)	Percent degradation (%) of WEO^a^	
					Observed	Predicted
1	34	6	5	42.63	26.32	26.68
2	40	6	5	40.2	16.14	15.12
3	34	8	5	52.5	40.36	41.88
4	40	8	5	50.24	32.43	30.32
5	34	7	3	54.75	42.5	43.26
6	40	7	3	50.5	32.67	31.7
7	34	7	7	55	42.43	39.10
8	40	7	7	41	24.31	27.54
9	37	6	3	52.9	34.25	32.30
10	37	8	3	58.63	45.34	47.5
11	37	6	7	50	26.25	28.15
12	37	8	7	60.52	45.7	43.34
13	37	7	5	72.4	56.21	55.08
14	37	7	5	70.67	54.25	55.08
15	37	7	5	71.68	55.43	55.08

^a^All values are represented as (mean ± SD) based on triplicate assays

A 3^3^ Box–Behnken experimental design was carried out with three replicates at the center points leading to 15 runs. The variables were coded according to the following equation:

1 $$ x_{i} = \, \left( {X_{i} {-} \, X_{0} } \right) \, / \, \Delta X\quad i \, = \, 1,2,3 \ldots k $$

where *x*_*i*_ is the dimensionless value of an independent variable, *X*_*i*_ is the real value of an independent variable, *X*_0_ is the value of *x*_*i*_ at the center point, and ∆*X* is the step change value.

The second-order polynomial model was fitted to response giving an equation term:

2 $$ \widetilde{Y}_{i} = \, \beta_{0} + \, \sum\limits_{i = 1}^{3} {\beta_{i} x_{i} } + \, \sum\limits_{i = 1}^{3} {\beta_{ii} x_{i}^{2} } + \, \sum\limits_{i,j = 1}^{3} {\beta_{ij} x_{i} x_{j} } $$

where ${{\tilde{\rm Y}}}_{i}$ is the predicted response, *x*_*i*_ and *x*_*j*_ are the input variables, *β*_0_ is the intercept term, *β*_*i*_ is the linear effects, *β*_*ii*_ is the squared effects, and *β*_*ij*_ is the interaction term.

‘Statistica v. 10’ software was used for regression and graphical analysis of the data. The optimum values of culture condition variables (pH, temperature and oil concentration) were obtained by solving the regression equation and analyzing the response surface contour plots.

## Results and discussion

### Isolation and identification of microorganism

**Fig. 1:**
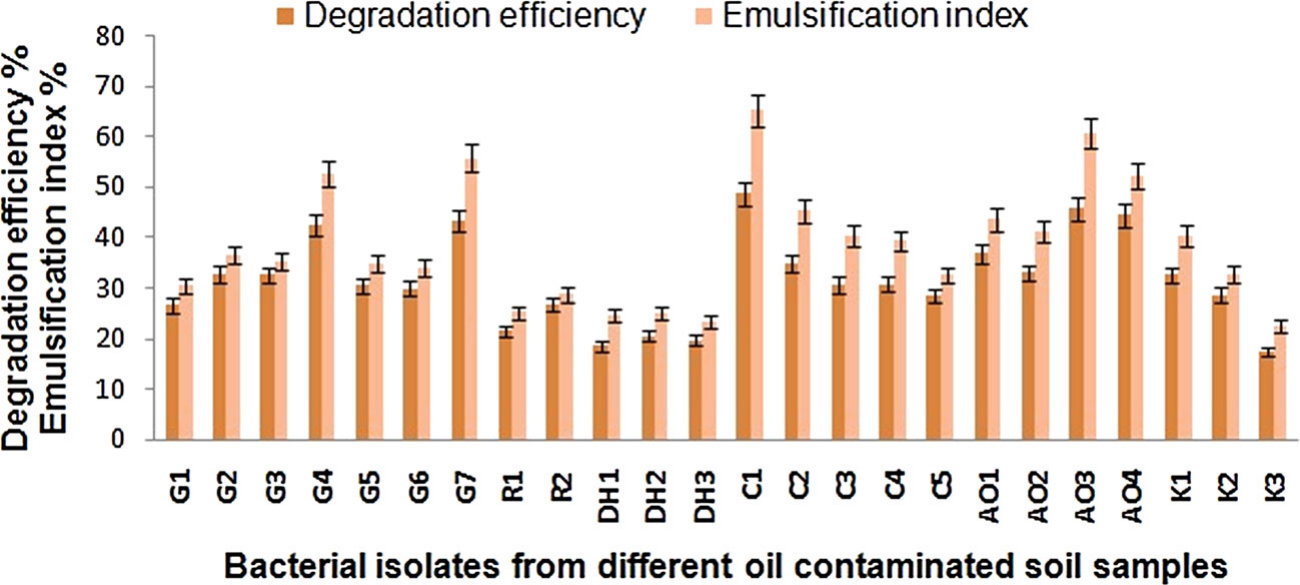
Screening of waste lubricant degrader microorganism from different oil-contaminated soil samples

**Fig. 2:**
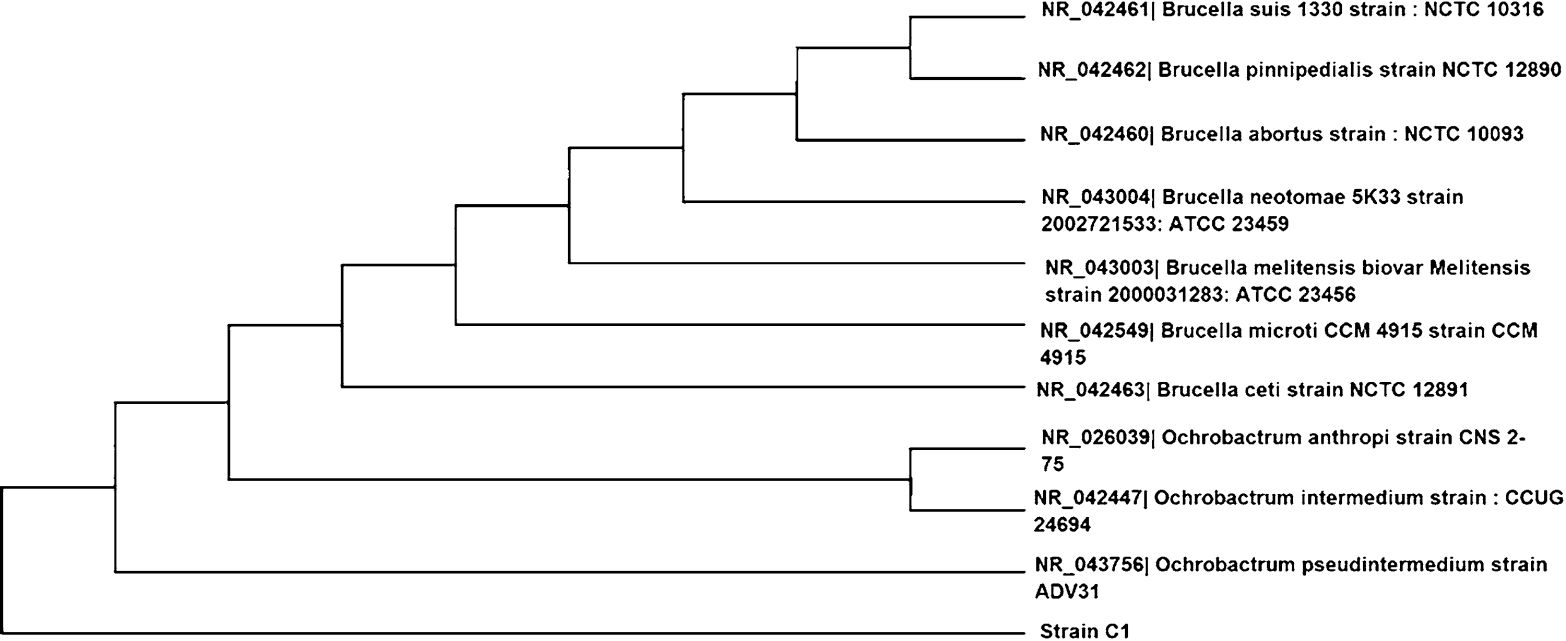
Phylogenetic tree showing interrelationship of isolated strain C1 with closely related species of the different genera inferred from 16S rRNA sequences. The tree was generated using the Neighbor-Joining method

Twenty-four bacterial strains were isolated and tested for waste lubricating oil degrading ability and emulsification ability (Fig. 1) among which only strain C1 was selected for its best degrading efficiency of WEO and *E*_24_ values using engine oil as substrate. This strain is an aerobic, gram-negative short rod. Sequence analysis of the 16S rRNA gene revealed 93 % homology with *Ochrobactrum pseudintermedium* strain ADV31. The phylogenetic tree generated using Neighbor-Joining method was provided in Fig. 2. The sequence has been submitted to GenBank with the accession no. KJ094035. Thus, our isolated strain C1 should be classified as a strain of *Ochrobactrum* sp. C1. The genus *Ochrobactrum* has been reported to degrade phenanthrene (Ghosal et al. 2010), phenol (Kılıç 2009), nicotine (Yuan et al. 2006), methyl parathion (Qiu et al. 2007), and various hydrocarbons (Calvo et al. 2008). Our study extended its application to the degradation of waste lubricating oil.

### Degradation characteristics of the strain C1

#### Biodegradability of hydrocarbons of N–P–A series

**Fig. 3:**
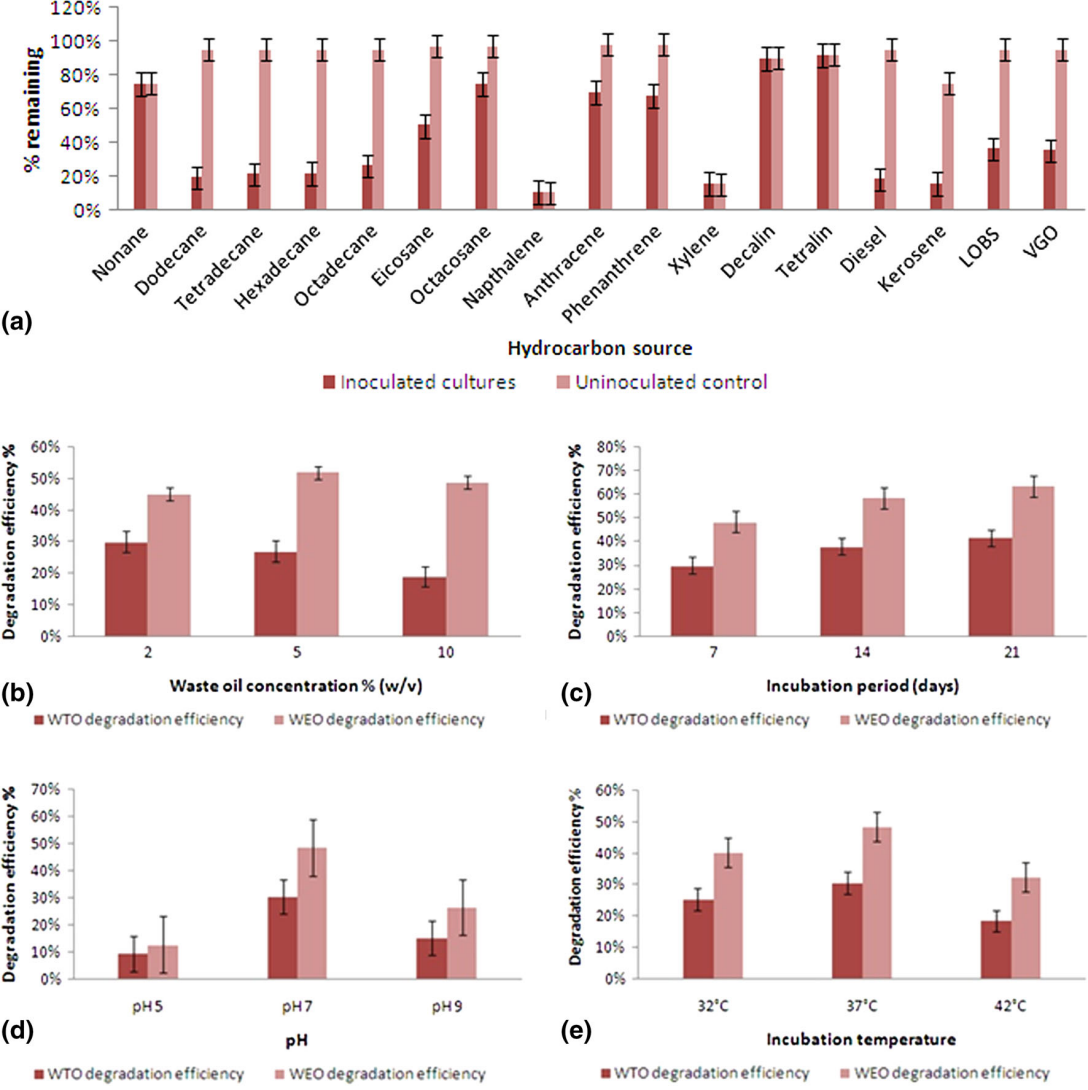
Hydrocarbon degradation characteristics and degradation efficiency of WEO and WTO by *Ochrobactrum* sp. C1 with 2 % (v/v) initial concentration of carbon source at pH 7.0, temperature 37 °C and 7 days incubation period. **a** Degradation percentage of different hydrocarbons; **b** effect of initial waste oil concentrations on percent degradation of WEO and WTO (at constant temperature, pH and incubation period); **c** effect of incubation period on percent degradation of WEO and WTO (at constant pH, temperature and waste oil concentration); **d** effect of initial medium pH on percent degradation of WEO and WTO (at constant temperature, incubation period and waste oil concentration); **e** effect of incubation temperature on percent degradation of WEO and WTO (at constant pH, incubation period and waste oil concentration); *Error bars*, mean ± SD of three replicates

The hydrocarbon utilization was performed with various hydrocarbon substrates to find out some insight about the substrate specificity for hydrocarbon biodegradation by *Ochrobactrum* sp. C1 as shown in Fig. 3a. The strain could metabolize linear *n*-alkanes ranging from C_12_–C_28_, but degradation efficiency was found to be decreased as alkane chain length increased as reported by several other researchers (Wang et al. 2011; Zhang et al. 2011). The losses affected by abiotic process during the incubation period were greater with the lower molecular mass alkanes than the alkanes of longer chain length. Among the aromatics, xylene and naphthalene were vaporized during period of incubation and the strain was only found to metabolize the PAHs when grown with phenanthrene and anthracene. Degradation efficiency was also higher with n-alkanes than the PAHs as represented in Fig. 3a. The strain could not metabolize decalin and tetralin from naphthene series probably due to microbial toxicity of these compounds (Sikkema et al. 1995).

#### Biodegradability of petroleum fractions and synthetic lubricants

More than 70 % degradation of the petroleum fractions was observed (Fig. 3a), which suggests that the strain is very much efficient for biodegradation of waste petroleum. Selective degradation of synthetic lubricants was also observed by the strain within 7 days incubation period. The lubricant removal efficiency of each strain was influenced by the types of lubricant. Montagnolli et al. (2009) reported that mineral lubricant is the most biodegradable, followed by semi-synthetic and synthetic lubricant.

### Waste lubricant degradation in pure culture

The initial concentrations of WEO and WTO used for biodegradation studies were 2 % (v/v), i.e., 17,200 and 16,300 mg L^−1^, respectively. Percentage degradation by *Ochrobactrum* sp. C1 was found to be 48.5 ± 0.50 % for WEO and 30.47 ± 0.25 % for WTO at temperature 37 °C, pH 7 and 7 days incubation period. During the incubation period, an emulsification of the WEO was observed in the culture broth, which suggests that the production of extra cellular bioemulsifier may be one of the mechanisms used by the present isolate in the utilization of WEO. Obayori et al. (2009) also reported similar type of emulsification capability of the culture supernatant by *Pseudomonas* sp. LP1 when grown on waste motor lubricating oil. The cell-free culture supernatant showed no significant reduction in surface tension of water (72 ± 0.15 to 55 ± 0.27 mN/m), but it showed emulsification ability toward various oils including kerosene, diesel, crude oil and engine oil. Emulsification index was found to be much higher (*E*_24_ 65 ± 0.32 %) using engine oil as substrate.

#### Effect of oil concentration

The degradation of WEO and WTO at various initial levels (2, 5 and 10 % v/v) was examined in BH medium at pH 7.0 and 37 °C over 7 days incubation period (Fig. 3b). No significant reduction in oil was observed for all controls. The percentage degradation was 48.83 ± 0.31, 52.3 ± 0.36 and 45.5 ± 0.40 %, respectively, for WEO. The result is in agreement with the findings of Abioye et al. (2012) who reported 3–6 % as the concentration of used motor oil for maximum rate of biodegradation. For WTO, the percentage degradation was 30.43 ± 0.80, 27.32 ± 0.64 and 18.74 ± 0.60 %, respectively. It was observed that the percentage degradation of WTO was decreased with increasing oil concentration and was much lower than the degradation efficiency of WEO, possibly due to the presence of highly persistent chlorinated alkanes as reported by Molnar et al. (2005).

#### Effect of incubation period

The effect of increasing incubation period from 7, 14 and 21 days was checked to find out the maximum extent of degradation efficiency at same culture parameters with 2 % (v/v) waste oil concentrations. It was found that percentage degradation increased from 48.35 ± 0.37 to 63.5 ± 0.50 % for WEO and 30.74 ± 0.42 to 41.5 ± 0.48 % for WTO on increasing the incubation period from 7 to 21 days (Fig. 3c). Bacterial growth is slower on insoluble hydrocarbon substrates due to less bioavailability, and it is one of the major constraints in bioremediation experiments. Several other researchers (Ghazali et al. 2004; Wang et al. 2011; Abioye et al. 2012) also found that TPH levels could be significantly reduced with longer incubation period during treatment of waste oil-contaminated soil. Biswal et al. (2009) reported that 40–50 % degradation of diesel engine lubricating oil could be achieved within 7 days.

#### Effect of pH

The degradation of WEO and WTO at various initial pH levels (5, 7 and 9) were tested in BH medium with 2 % (v/v) oil concentration at 37 °C over 7 days incubation period (Fig. 3d). As shown, when the pH was 5.0, 7.0 and 9.0, percentage degradation of WEO were 12.82 ± 0.24, 48.56 ± 0.37, 26.54 ± 0.20 % and percentage degradation of WTO were 9.37 ± 0.30, 30.63 ± 0.41, 15.42 ± 0.26 %, respectively. Only abiotic loss was observed for all controls. The results indicated that near-neutral condition was favorable for the degradation of waste lubricants by strain C1, whereas higher or lower pH inhibited degradation. The results were consistent with the findings of Jain et al. (2010), who also found that near neutral pH was most favorable for degradation of petroleum hydrocarbons in crude oil.

#### Effect of temperature

The effect of incubation temperature on biodegradation of WEO and WTO in BH medium with 2 % (v/v) oil concentration, pH 7.0 and 7 days incubation period. Abiotic percentage loss of waste oils was observed for all controls. As shown in Fig. 3e, percentage degradation of WEO was 40.34 ± 0.27, 48.46 ± 0.32 and 32.51 ± 0.30 % for 32, 37 and 42 °C, respectively, whereas percentage degradation of WTO were found to be 25.35 ± 0.34, 30.57 ± 0.29 and 18.52 ± 0.31 % for temperatures 32, 37 and 42 °C, respectively. The results indicated that the optimum temperature for biodegradation of waste lubricants by strain C1 in pure culture was 37 °C. Aleer et al. (2011) also found that 30–37 °C was the optimum temperature for WEO biodegradation by a microbial consortium.

### Optimization of culture conditions

The effects of three variables viz. pH, temperature and oil concentration on percent degradation of WEO were studied to achieve optimum biodegradation within 7 days incubation period. The optimum values were determined using the Box–Behnken experimental design matrix presented in Table 1. Analyzing these data by multiple regressions, the following quadratic polynomial equation was found to express the percentage degradation of WEO:

3 $$ \widetilde{Y}_{i} = \, 33.91 \, {-} \, 10.71X_{1} + \, 15.13X_{2} {-} \, 4.23X_{3} + \, 14.5X_{1}^{2} + \, 12.08X_{2}^{2} + \, 5.18X_{3}^{2} + \, 1.00X_{1} X_{2} {-} \, 4.3X_{1} X_{3} + \, 4.31X_{2} X_{3} $$

where ${{\tilde{\rm Y}}}_{i}$ is the predicted percent degradation, *X*_1_ temperature, *X*_2_ pH and *X*_3_ waste oil concentration.

ANOVA results of the second-order response surface model summarized in Table 2. Fischer’s *F* test was performed on experimental data to evaluate the statistical significance of the model. According to ANOVA, the *F* values for all regressions were high, indicated that most of the variations on the response variables can be explained by the regression equation. A *p* value lower than 0.05 indicated that the model is considered to be statistically significant (Myers and Montgomery 2002). Goodness of fit was examined by the correlation coefficient (*R* ^2^ = 0.998) between the experimental and modeled data represented in Table 1, which revealed a linear mathematical relation among them confirming the adequacy of the regression model. The mathematical adjust of these values considering residual error generated a *R* ^2^ (adjusted) = 0.9943, revealing that only 0.57 % of the overall effects could not be explained by the model which again shows the model to be highly significant. In addition the mismatching analysis (*P*_lack of fit_ = 0.37) was observed to be insignificant, implying that the obtained model was adequate to explain the experimental data.

**Table 2:** Analysis of variance (ANOVA) for biodegradation of waste engine oil and model coefficients estimated by multiple linear regression analysis

Factor	Coefficient (std. error)	Sum of squares	*df* ^a^	*F* value^b^	Computed *t* value *t*(2)	*p* value* Prob > *F* ^c^
Intercept	33.91 (0.25)				133.78	0.000056*
*X*_1 _(L)	−10.71 (0.65)	277.662	1	360.210	−16.37	0.002765*
*X*_1_^2^ (Q)	15.13 (0.65)	776.620	1	1,007.507	23.12	0.000991*
*X*_2_ (L)	−4.23 (0.65)	419.345	1	544.015	−6.46	0.001833*
*X*_2_^2^ (Q)	14.5 (0.46)	538.842	1	699.039	31.74	0.001427*
*X*_3_ (L)	12.08 (0.46)	30.062	1	38.999	26.44	0.024696*
*X*_3_^2^ (Q)	5.18 (0.46)	99.089	1	128.548	11.34	0.007690*
*X*_1_ *X*_3_ (1L by 3L)	1.00 (0.88)	18.490	1	23.987	1.14	0.039251*
*X*_1_ *X*_2_ (1L by 2L)	−4.3 (0.88)	13.158	1	17.070	−4.89	0.053889
*X*_2_ *X*_3_ (2L by 3L)	4.31 (0.88)	18.533	1	24.043	4.9	0.039165*
2L by 3Q		0.078	1	0.101		0.780534
2Q by 3L		0.090	1	0.117		0.764757
Lack of fit		1.000	1	1.297		0.372750
Pure error		1.542	2			
Total SS		2,080.269	14			

*R* ^2^ = 0.99878

Adjusted *R* ^2^ = 0.9943

* *p* < 0.05 are considered to be significant

^a^Degree of freedom

^b^Test for comparing model variance with residual (error) variance

^c^Probability of seeing the observed *F* value if the null hypothesis is true

The significance of each coefficient of the model was determined by Student’s *t* test and *p* value (Table 2). Larger magnitude of *t* test and smaller *p* value was indicative of a more significant coefficient effect (Myers and Montgomery 2002). It was observed that the linear and quadratic effects of all three parameters and the interaction effects between temperature–oil concentration and pH–oil concentration were significant with a *p* value <0.05.

**Fig. 4:**
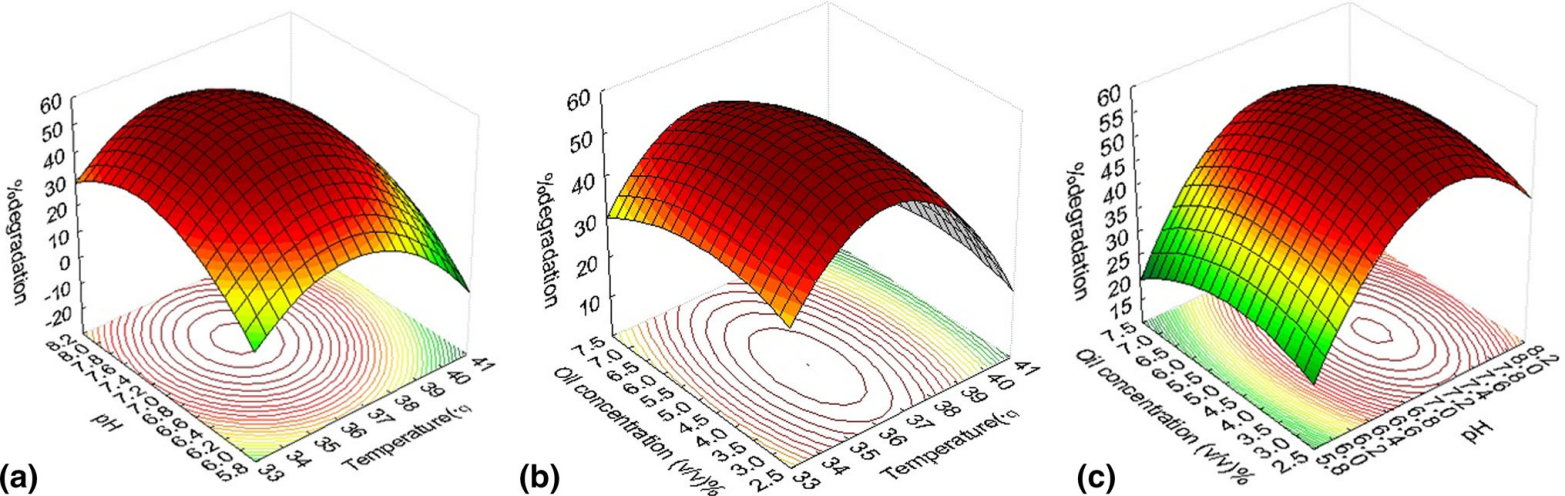
Response surface plots for degradation of WEO by *Ochrobactrum* sp. C1 **a** effects of temperature and pH; **b** effects of temperature and oil concentration; **c** effects of oil concentration and pH

The 3D response surface plots described by the regression model were drawn to illustrate the effects of the independent variables, and combined effects of each independent variable upon the response variable. Figure 4a–c represents the 3D response surface plots for the optimum culture conditions of waste engine oil degradation. Each figure presents the effect of two variables, while the third was held at zero. To determine the optimized culture condition, the optimal values of the variables *X*_1_, *X*_2_ and *X*_3_ were found out by solving the regression equation. This was achieved by putting the second-order regression equation into matrix form as described by Myers and Montgomery (2002). The model predicted that with temperature 36.4 °C, pH 7.3 and waste oil concentration 4.6 % (v/v), the maximum percent degradation rate will be 57 %.

### Biodegradation of WEO at optimized culture conditions

**Fig. 5:**
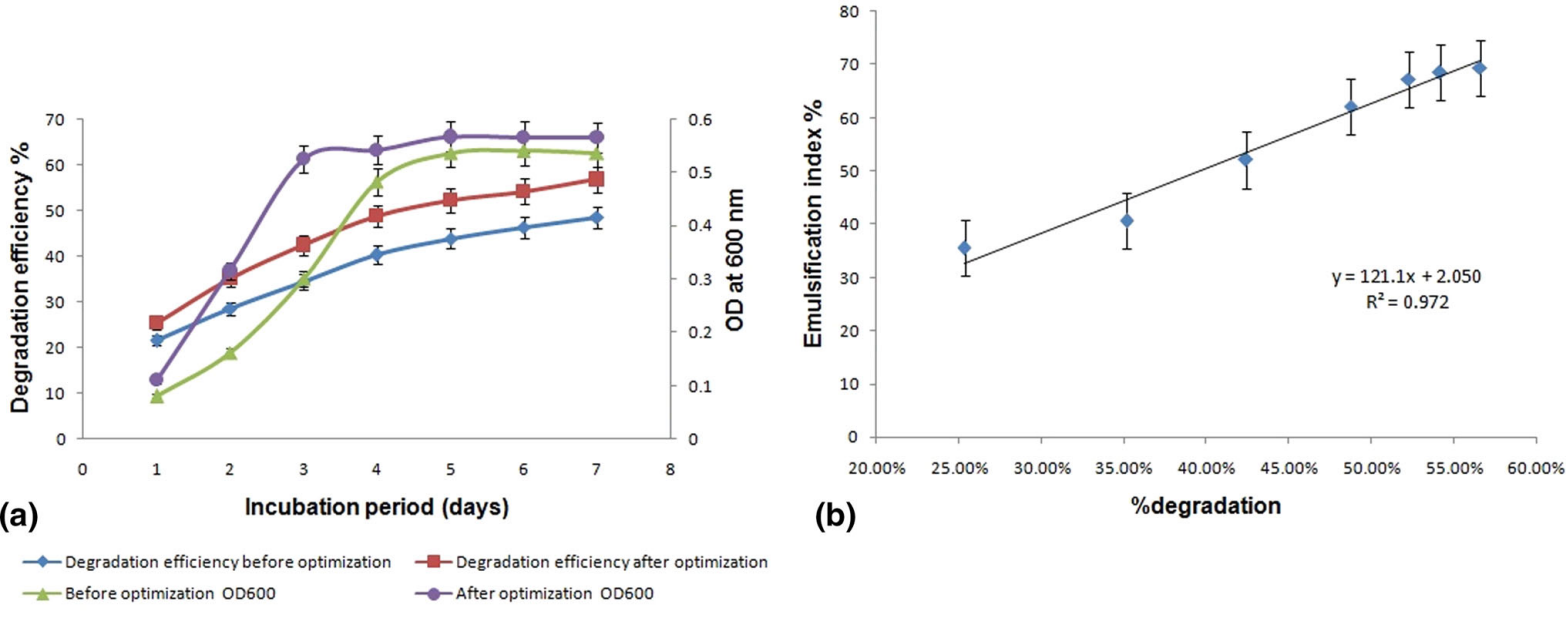
Validation experiment for biodegradation of WEO **a** degradation efficiency and culture density before and after optimization by *Ochrobactrum* sp. C1 over incubation period of 7 days; **b** correlation between percent degradation and emulsification index of culture broth; *Error bars*, mean ± SD of three replicates

Subsequent experiments with the optimized culture condition yielded significant results of 56.7 ± 0.25 % removal of WEO with an initial concentration of 4.6 % (v/v), i.e., 39,560 mg L^−1^ within 7 days, which is also consistent with the prediction. Figure 5a showed the percentage degradation of WEO and bacterial culture density before and after optimization of culture parameters over 7 days incubation period, which indicates positive enhancement of growth and biodegradation efficiency of the strain *Ochrobactrum* sp. C1., whereas Fig. 5b represented a good correlation between rate of percent degradation and emulsification activity (highest *E*_24_ = 69.42 ± 0.32 %) of the culture broth with a correlation coefficient *r* = 0.972, indicating that the uptake of waste oil might be enhanced due to production of emulsifying agent by *Ochrobactrum* sp. C1. The production, isolation and characterization of the emulsifying agent have also been reported previously by Bhattacharya et al. (2014b). Similar findings were also reported by Mehdi and Giti (2008) about correlation between biofilm formation, biosurfactant production and crude oil biodegradation. Hua and Wang (2012) also found a positive correlation exists between surface-active agent production and uptake of octadecane by *Pseudomonas* sp. DG 17.

### Characterization of waste lubricating oils

**Fig. 6:**
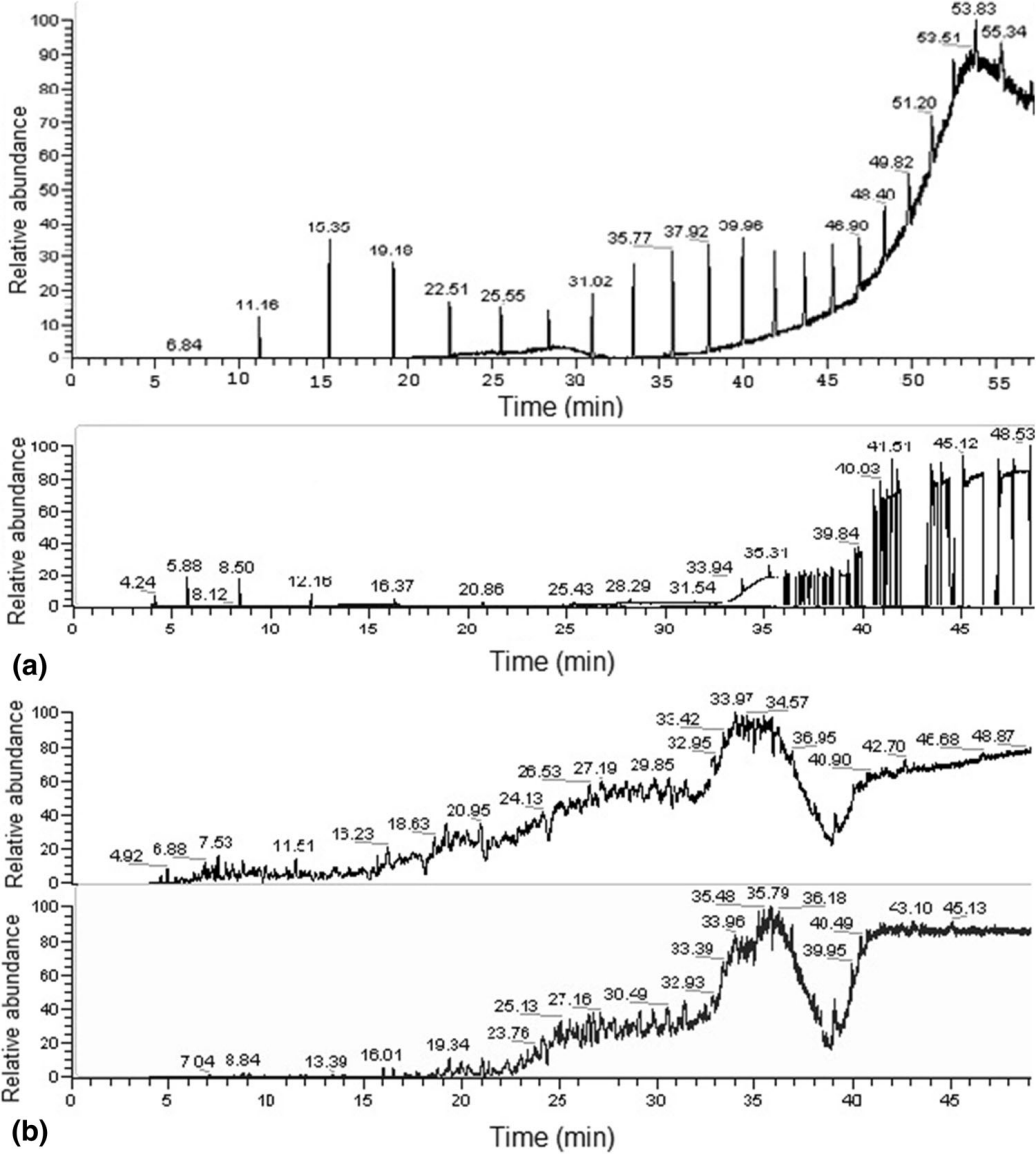
GC chromatogram of waste oil samples. **a** WEO residual oil sample extracted before and after biodegradation experiment; **b** WTO residual oil sample extracted before and after biodegradation experiment

Waste lubricating oils may have highly variable compositions according to the extent of combustion process during its functioning, so the biodegradation analysis was done by comparing the content of recoverable total petroleum hydrocarbons (TPH) from influent and effluent oil samples. The GC spectra of hydrocarbon compounds occurring in both the treated and untreated waste oil samples, i.e., WEO and WTO, were expressed in Fig. 6a, b. Both the waste oil samples after biological treatment, revealed significant reduction in major hydrocarbon peaks compared to those from untreated control samples. The most abundant peaks from WEO were identified as derivatives of benzene, naphthalene, azulene, indole, benzopyrene, dibenzophenazine, etc., whereas the compounds identified from WTO sample, mostly consisted biphenyl and naphthene derivatives. Benzene-based compounds and naphthalene-related components are the predominant hydrocarbon structures in the composition of both the waste lubricating oil samples. Naphthalene, acenaphthylenes, diphenylanthracenes, benzopyrene and benzanthracene were also detected in used motor oil samples by several other researchers (Koma et al. 2003; Wang et al. 2010). In addition, it was also observed from Fig. 6 that low molecular weight benzene derivatives were better removed by strain C1 than the high molecular weight benzene derivatives according to their relative abundance, in which the point was also supported by Genov et al. (2008).

## Conclusion

A newly isolated *Ochrobactrum* sp. C1 could grow with waste lubricants as the sole carbon and energy source and degrade a wide range of hydrocarbons present in waste lubricants efficiently. The culture parameters were optimized using response surface methodology and the optimized culture condition (temperature 36.4 °C, pH 7.3 and 4.6 % (v/v) oil concentration) yielded significant results of 56.7 ± 0.25 % removal of WEO within 7 days. Moreover, the culture showed high emulsification capability toward a range of hydrocarbons, among which the highest emulsification index (*E*_24_ = 69.42 ± 0.32 %) was obtained with engine oil after 7 days incubation period at optimized culture condition. Therefore, biodegradation and optimization studies with *Ochrobactrum* sp. C1 may be of great significance in bioremediation of highly contaminated soils or sewage sludge with waste lubricants in bioreactor systems.
